# Simultaneous C5 and CD14 inhibition limits inflammation and organ dysfunction in pig polytrauma

**DOI:** 10.3389/fimmu.2022.952267

**Published:** 2022-08-18

**Authors:** Ludmila Lupu, Klemens Horst, Johannes Greven, Ümit Mert, Judith A.K. Ludviksen, Kristin Pettersen, Corinna Lau, Yang Li, Annette Palmer, Kang Qin, Xing Zhang, Benjamin Mayer, Martijn van Griensven, Markus Huber-Lang, Frank Hildebrand, Tom Eirik Mollnes

**Affiliations:** ^1^ Institute of Clinical and Experimental Trauma Immunology, University Hospital Ulm, Ulm, Germany; ^2^ Department of Orthopedics, Trauma and Reconstructive Surgery, Rheinisch-Westfalische Technische Hochschule (RWTH) Aachen University, Aachen, Germany; ^3^ Research Laboratory, Nordland Hospital Bodø, Bodø, Norway; ^4^ Institute of Epidemiology and Medical Biometry, Ulm University, Ulm, Germany; ^5^ Department Cell Biology-Inspired Tissue Engineering (cBITE), MERLN Institute for Technology-Inspired Regenerative Medicine, Maastricht University, Maastricht, Netherlands; ^6^ Department of Immunology, Oslo University Hospital, and University of Oslo, Oslo, Norway; ^7^ Center of Molecular Inflammation Research, Norwegian University of Science and Technology, Trondheim, Norway

**Keywords:** complement, CD14, TLR, inflammation, trauma and MODS

## Abstract

Dysfunctional complement activation and Toll-like receptor signaling immediately after trauma are associated with development of trauma-induced coagulopathy and multiple organ dysfunction syndrome. We assessed the efficacy of the combined inhibition therapy of complement factor C5 and the TLR co-receptor CD14 on thrombo-inflammation and organ damage in an exploratory 72-h polytrauma porcine model, conducted under standard surgical and intensive care management procedures. Twelve male pigs were subjected to polytrauma, followed by resuscitation (ATLS^®^ guidelines) and operation of the femur fracture (intramedullary nailing technique). The pigs were allocated to combined C5 and CD14 inhibition therapy group (n=4) and control group (n=8). The therapy group received intravenously C5 inhibitor (RA101295) and anti-CD14 antibody (rMil2) 30 min post-trauma. Controls received saline. Combined C5 and CD14 inhibition reduced the blood levels of the terminal complement complex (TCC) by 70% (p=0.004), CRP by 28% (p=0.004), and IL-6 by 52% (p=0.048). The inhibition therapy prevented the platelet consumption by 18% and TAT formation by 77% (p=0.008). Moreover, the norepinephrine requirements in the treated group were reduced by 88%. The inhibition therapy limited the organ damage, thereby reducing the blood lipase values by 50% (p=0.028), LDH by 30% (p=0.004), AST by 33%, and NGAL by 30%. Immunofluorescent analysis of the lung tissue revealed C5b-9 deposition on blood vessels in five from the untreated, and in none of the treated animals. In kidney and liver, the C5b-9 deposition was similarly detected mainly the untreated as compared to the treated animals. Combined C5 and CD14 inhibition limited the inflammatory response, the organ damage, and reduced the catecholamine requirements after experimental polytrauma and might be a promising therapeutic approach.

## Introduction

Severely injured patients frequently develop complications, including coagulopathy, sepsis and multiple organ dysfunction syndrome (MODS) (1). The underlying mechanisms of posttraumatic thrombo-inflammation and organ dysfunction are driven by the trauma-induced immune response (2), which is regulated mainly by the complement cascade and the Toll-like receptor (TLR) signaling pathway (2).

The complement cascade is activated within minutes after trauma (3), and reaches its peak activity within the first 6-h post-injury (4). Complement was reported to be responsible for the oxidative burst in granulocytes and monocytes (5, 6), contributing to development of lung and kidney dysfunction after trauma (7, 8). C5a-primed neutrophils augment their production of reactive oxygen species once exposed to a “second-hit” traumatic event, such as surgery, increasing the risk for thromboembolic events (6).

The CD14 antigen is a cofactor of several TLRs, and plays a crucial role in pathogen-associated molecular patterns (PAMPs) and damage-associated molecular patterns (DAMPs) recognition (9). In a recent study, single-cell mass cytometry analysis applied to blood samples from patients with hip-replacement surgery, revealed that the prolonged postoperative recovery, pain, and functional impairment were strongly correlated with the cell signaling responses of CD14^+^ cells (10). In trauma settings, the activation of TLRs was associated with prothrombotic events and increased thrombin-antithrombin (TAT) complexes in blood (11). Furthermore, posttraumatic TLRs signaling pathway activation has been associated with lung and kidney dysfunction (12, 13).

Both complement system activation and CD14 antigen expression pattern are associated with monocyte exhaustion in sterile and infection-induced inflammation (14, 15), with life-threatening consequences for the patients. Single-cell RNA-sequencing analysis of murine monocytes revealed that a subclinical LPS dosage could program the monocytes into low-grade inflammatory state, with elevated level of chemokines, chemokines receptors, and C5aR1 expression (16). A subset of CD14^+^ cells was shown to distinctly change its transcriptional state in bacterial sepsis (17), in severe Covid-19 infection (18), and also in sterile infection caused by trauma, thus delaying the recovery and increasing the likelihood of developing complications (19).

Therefore, inhibition of excessive complement and TLR activation might be beneficial in the posttraumatic immune response. Although, the concept of combined inhibition in sepsis was extensively studied (20–25), this regimen has never been tested in polytrauma. Consequently, the aim of this study was to investigate the effects of simultaneous inhibition of the complement cascade at the C5 level and of the TLR signaling pathway at the CD14 level in a long-term polytrauma porcine model. We hypothesize that combined C5 and CD14 inhibition therapy will improve the systemic inflammatory response, and thus prevent the development of the posttraumatic coagulopathy and MODS.

## Materials and methods

### Animals and experimental design

The well-established 72-h porcine polytrauma including hemorrhagic shock model (26) was approved by the responsible government authority (Ministry for nature, environment and consumer protection in North Rhine-Westphalia, Recklinghausen, Germany; AZ 81-02.04. 2020.A215). The study was performed in compliance with the German legislation, the Federation of European Laboratory Animal Science Association (FELASA), and the ARRIVE guidelines (27).

Twelve German landrace male pigs (Sus scrofa, aged 12–16 weeks, mean body weight 35 ± 5 kg) from a disease-free barrier breeding facility were housed in ventilated rooms and allowed to acclimatize to their surroundings for a minimum of 7 days before surgery. 12-h before the experiment, the animals were exposed to fasting with water *ad libitum*. Preparation and instrumentation were described in detail by Horst et al. (26). Briefly, animals were premedicated (Azaperone (Stresnil™, Janssen, Germany) in combination with Ketamine (Ketanest, Pfizer, New York)), intubated and induced in general anesthesia (Propofol (Fresenius, Germany) in combination with Midazolam (Panpharma GmbH, Germany)). Fentanyl (Panpharma GmbH, Germany) was used as a systemic analgesic. The general anesthesia and analgesia, as well as a lung protective ventilation strategy with a tidal volume of 8-12 ml/kg body weight (Evita 4, Draeger, Luebeck, Germany) were maintained throughout the entire experiment. The experimental animals were exposed to polytrauma: blunt chest trauma, laparotomy with liver laceration with a subsequent hemorrhagic shock, and bilateral open shaft femur fracture. The blunt chest trauma was induced using a bolt gun (Dynamit-Nobel, Vienna, Austria). A midline laparotomy was performed to expose the left liver lobe, on which two incisions (4.5cm × 4.5cm) were performed. The bilateral shaft femur fracture was performed using the bolt gun. Meanwhile, the pressure-controlled hemorrhagic shock was initiated by withdrawing blood from the femoral vein until the mean arterial pressure (MAP) fell to 40 ± 5mmHg and maintained for 90 min. Afterwards, the animals were resuscitated according to established trauma guidelines (ATLS^®^ guidelines). Throughout 72-h of experiment, the animals were given fluids (Sterofundin, B. Braun, Germany) at a rate of 0.5-2.0 ml/kg/h and parenteral nutrition (Aminoven, Fresenius Kabi, Germany) 370 kcal/l 50-70 ml/kg body weight and day, under a close monitoring of the fluid balance. The heart rate and MAP were monitored using a Philips patient monitor (Philips Health Systems, Hamburg, Germany). The femur fracture was reduced and fixed by an intramedullary nailing technique (Stryker, Duisburg, Germany). For supporting the respiratory mechanics of pressure-controlled ventilated pigs, the animals were turned every 4-6 hours. If required, norepinephrine was administered i.v. for maintaining the MAP>60 mmHg.

As numerous *in vitro* whole blood studies and *in vivo* mice studies showed the superiority of the combined inhibition therapy over the monotherapy (21–24), for the present study, the decision was taken to proceed only with combined inhibition therapy. This decision was also based on the experience form the previous large animal (pig) study of peritonitis-induced sepsis, where the ethical guidelines (the 3 Rs’) were closely followed, including saving the number of animals (25). The experimental animals were allocated to two groups: C5 and CD14 inhibition treated group (n=4) and untreated group (n=8). The treated group included 4 animals due to the limited access to the C5 inhibitor. The combined inhibition therapy included the intravenous (i.v.) administration of the C5 and CD14 inhibitors. One animal from the treated group was given a bolus of C5 inhibitor (3mg/kg) 30 min post-injury with a subsequent continuous infusion (0.55 mg/kg/h) until 64-h after the trauma, and one bolus of anti-CD14 (5mg/kg) at 30 min post-injury. The other three animals of the treated group received higher dosages of the combined inhibition therapy, based on the experiments testing the effects of the two inhibitors in the first animal. The final doses were bolus of C5 inhibitor (5mg/kg) 30 min post-trauma with a subsequent continuous infusion (1.1 mg/kg/h) until 72-h post-injury, and anti-CD14 boluses of 5mg/kg at 30 min, 12-, 30-h and of 2.5 mg/kg at 60-h post-injury. The four pigs were merged to one group since there was negligible variability in the treatment efficacy between the first and the other three pigs (p<0.05 for all parameters both for 3 vs 8 and 4 vs 8 pigs).

### Interventional drugs

RA101295 (2-kDa peptide) was provided by UCB Pharma (Brussels, Belgium). RA101295 is a C5 inhibitor, with a cross-species activity, that prevents C5 cleavage and the subsequent generation of C5a and formation of the terminal C5b–9 complement complex (TCC), which is present in two forms: as sC5b–9 in the fluid-phase and as the membrane attack complex (MAC) on the cell surface (28).

rMil2 is a recombinant anti-porcine CD14 antibody (clone MIL2; isotype IgG2a), made recombinant as an IgG2/4 chimera in the laboratory of Professor T.E. Mollnes (Norway) (29) and produced largescale by ExcellGene SA (Monthey, Switzerland) according to GMP standards. The anti-CD14 antibody blocks the CD14 antigen and has no effector functions (29). The CD14 antigen in pigs is located mainly on granulocytes and functions as a co-factor for TLR signaling (9, 29, 30).

### Immune monitoring of the combined inhibition therapy

**Figure 1:**
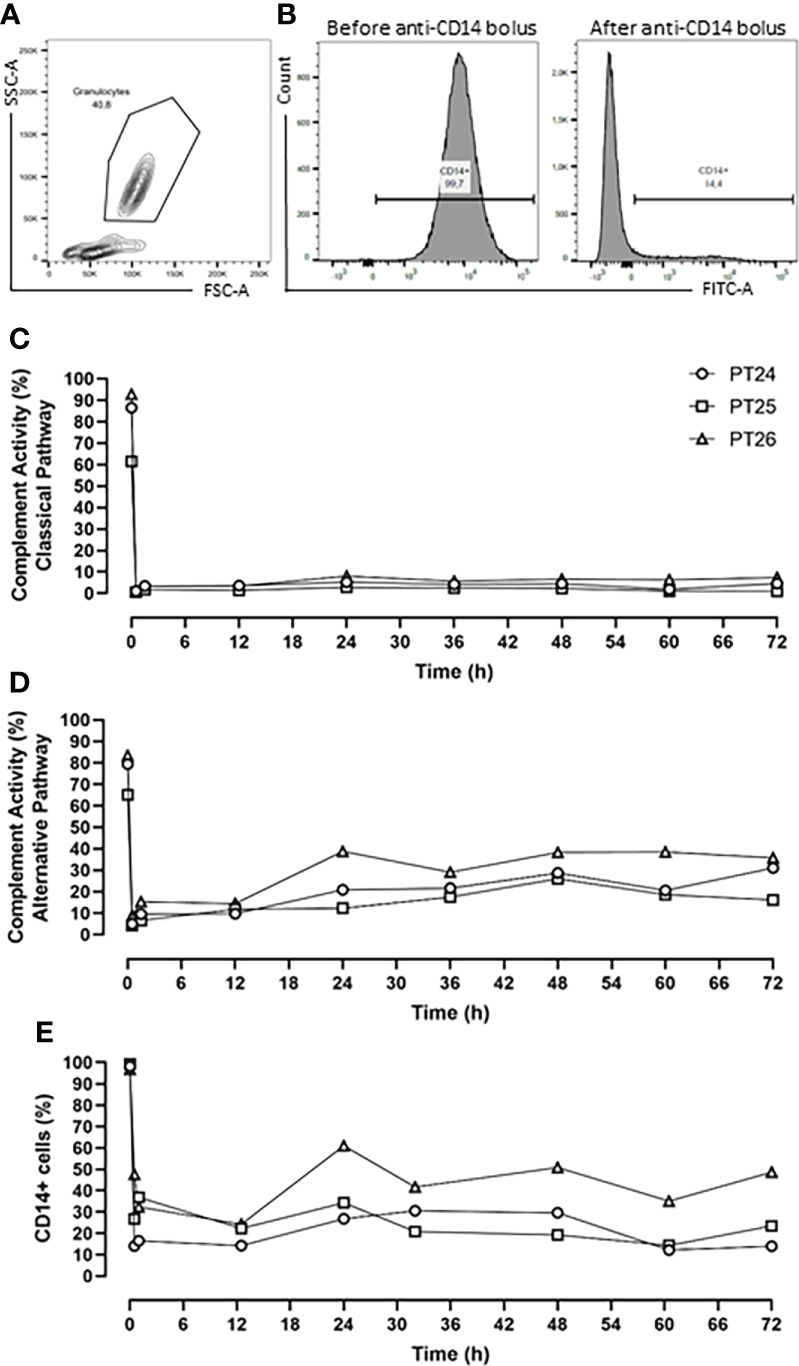
*Pharmacodynamic analysis.* **(A)** Flow Cytometry gating strategy of granulocyte. **(B)** Percentage of CD14+ cells before and after anti-CD14 (rMil2) application. **(C)** RA101295 (5mg/kg bolus, followed by 1.1mg/kg/h continuous infusion) blocked the classical pathway by 96%, and **(D)** the alternative pathway by 81%. **(E)** Simultaneous application of rMil2 (5mg/kg bolus at 30 min, 12-, 30-h, and 2.5mg/kg at 60-h) ensured CD14 antigen saturation of 75%. PT 24, PT25 and PT26 indicate experimental animal code in the treatment group.

The pharmacodynamics of the combined inhibition therapy were assessed every 12-h. The RA101295 pharmacological effect of blocking the classical and alternative complement pathways was evaluated using the Complement System Screen Wieslab (Euro Diagnostica, Malmö, Sweden) according to the manufacturer’s instructions. CD14 antigen saturation with the rMil2 anti-CD14 antibody was assessed by flow cytometry analysis using FITC-labelled rMil2 anti-CD14 antibody. Briefly, 75µl of EDTA blood was incubated with anti-CD14-FITC antibody at a concentration of 375 µg/ml. The cells were analyzed using BD FACSCanto™ II. The data are presented as percentage of CD14-FITC positive cells (**Figures 1A, B**).

### TCC measurement

TCC assessment in EDTA-plasma was performed using the ELISA-based technique (lower detection limit: 0.156 CAU/ml) as previously described (31). Briefly, the capture antibody aE11 (mouse monoclonal anti-C9 neoepitope antibody) reacts with an epitope that is exposed in C9 only when C9 is activated and incorporated into the C5b-9 complex. aE11 has been described previously as an anti-human C9 neoepitope antibody with a strong cross-reactivity to pig (32).

### Biochemistry and hematology tests

Complete blood count for the assessment of the white blood cells (WBCs) and platelets (PLTs) was performed using hematology analyzer (Nihon Kohden, Japan). C-reactive protein (CRP), lipase, lactate dehydrogenase (LDH), and aspartate transaminase (AST) analyses were measured in serum by chemistry analyzer (Ortho-Clinical Diagnostics, NJ).

### Cytokines, hemostatic and organ damage markers

The immunoassays for these analyses were chosen based on previous assessment of the different commercially available kits (33). Porcine ELISAs were used to quantify the interleukin 6 (IL-6) (lower detection limit: 9.4 pg/ml) and tumor necrosis factor (TNF) (lower detection limit: 11.7 pg/ml) (R&D Systems, MN); plasminogen activator inhibitor-1 (PAI-1) (lower detection limit: 3.1 ng/ml) (Nordic BioSite AB, Taby, Sweden) and neutrophil gelatinase-associated lipocalin (NGAL) (lower detection limit: 4.0 pg/ml) (Abcam, Cambridge, UK). Interleukin 8 (IL-8) was measured in bronchoalveolar lavage fluid (BALF) using Multiplex analysis (lower detection limit: 12.0 pg/ml) (Merck, Darmstadt, Germany). Human ELISA, documented in T.E. Mollnes laboratory to cross-react with pigs, was used to measure the thrombin–antithrombin complex (TAT) (lower detection limit: 2.0 µg/ml) (Siemens Healthineers, Germany). All the assays were performed using snap-frozen EDTA-plasma in accordance with the manufacturers’ instructions.

### Microscopy analysis

Tissue-Tek OCT (Sakura Finetek, Torrance, CA) embedded lung, kidney, and liver cryosections (7µm) were incubated overnight at 4°C with primary antibody: mouse monoclonal anti-C9 neoepitope exposed in C5b-9, clone aE11 as described above (provided by Professor T.E. Mollnes (Norway)). Goat anti-mouse IgG2a conjugated with Alexa Fluor^®^ 568 (Invitrogen, MA) was used as secondary Ab. The F-actin was stained using Phalloidin Alexa Fluor^®^ 488 (Invitrogen). The slides were mounted with Vectashield mounting medium (Vector Laboratories, Burlingame, CA) containing DAPI for nuclear counterstain. The images were captured using a Zeiss Axio-Imager microscope (Jena, Germany). The images were analyzed based on the presence of absence of the fluorescent signal.

### Data presentation and statistics

The results from Wieslab Complement System Screening and Flow Cytometry analysis are given as individual profiles (n=3). GraphPad Prism version 9.2.0 (San Diego, CA) and the R software version 4.1.2 (www.r-project.org) were used for data visualization and statistical analysis. The latter was primarily based on non-parametric approaches because of the low sample sizes. Efficacy of the interventional drugs was evaluated by means of different ancillary approaches:

On the one hand, longitudinal measurements of the outcome parameters were initially summarized by time points and displayed using median values and corresponding interquartile ranges (IQR). The nonparametric Mann-Whitney-U test was used in order to compare treated and untreated groups at different time points. Further, to account for the repeated measurement structure of the data, a nonparametric approach of longitudinal data analysis in factorial designs was applied (R package nparLD) assessing the difference between treated and untreated animals (group changes over time) for the TCC, lipase, TNF, and NGAL data. Due to missing values, for the remaining variables (WBC, PLT, CRP, AST, LDH, TAT, PAI-1, IL-6) a mixed linear model analysis was applied, since the nparLD approach mentioned above requires complete data.

On the other hand, for each individual trajectory an area under the curve (AUC) was calculated. These individual AUCs reflect the development of each particular outcome parameter over time. All individual AUC values were then summarized using median (IQR) for each group of treated and untreated animals separately. Again, the Mann-Whitney-U test was used to compare the median AUCs between treated and untreated animals. The percentage change of the AUC, reported in the text, represents the difference between the AUC median values of the untreated and the treated group. These AUC results are thought to support the findings from longitudinal data analysis. In the AUC graph representation, the treated pig who received lower medication dosages is depicted as filled black symbol color.

Correlation analysis was performed using nonparametric Spearman rank correlation. For statistical analysis of the immunofluorescence staining results Fisher’s exact test was used. Statistical significance was assumed in case of a two-sided P-value < 0.05 (*: p<0.05, **: p<0.01, ***: p<0.001). Because of the study setting, all p-values are interpreted in an exploratory manner, which is why there was no correction of the type 1 error level due to multiple hypothesis testing.

One animal from the untreated group died at 60-h. It was included in the study analysis, while for the time point of 72-h the median of the other seven animals from the untreated group was calculated and used for the statistical analysis.

## Results

### Defining the dose of the drugs

The C5 inhibitor RA101295 injected i.v. 30 min post-injury as a bolus (3 mg/kg) with a subsequent continuous i.v. administration (0.55 mg/kg/h) until 64-h post-injury, blocked the classical complement pathway by 76% and the alternative pathway by 62% throughout the experiment (**Supplementary Figures 1A, B**). For optimizing the inhibitory effects, it was decided to increase the RA101295 dosage and duration of the administration for the next three animals. Therefore, RA101295 administration as a bolus of 5 mg/kg (30 min post-injury) with a subsequent continuous i.v. administration (1.1 mg/kg/h) until 72-h post-injury, significantly enhanced the blocking efficacy for the classical complement pathway to 96% and the alternative pathway to 81% throughout the experiment (**Figures 1C, D**).

Intravenous administration of a single bolus of the anti-CD14 antibody (5mg/kg) 30 min after trauma ensured a saturation of 79% of the CD14 antigen on the granulocytes throughout the experiment (**Supplementary Figure 1C**). However, the flow-cytometry-based immune monitoring, revealed a high granulocytes turnover after trauma and surgical management, consistent with the slight increase seen during the experiment in the first pig. Therefore, the anti-CD14 regimen was adjusted by giving the anti-CD14 antibody in boluses of 5mg/kg at 30 min, 12-, 30-h and of 2.5 mg/kg at 60-h post-injury, which ensured a continuously stable saturation of 75% of the CD14 antigen throughout the experiment (**Figure 1E**).

Despite a modest increase in the efficacy after dose adjustments, a substantial inhibition was observed in the first pig and the four pigs were therefore regarded as one single group with very similar treatment efficacy, as described statistically in the Materials and Methods section.

### Combined C5 and CD14 inhibition therapy reduced catecholamine requirement

**Figure 2:**
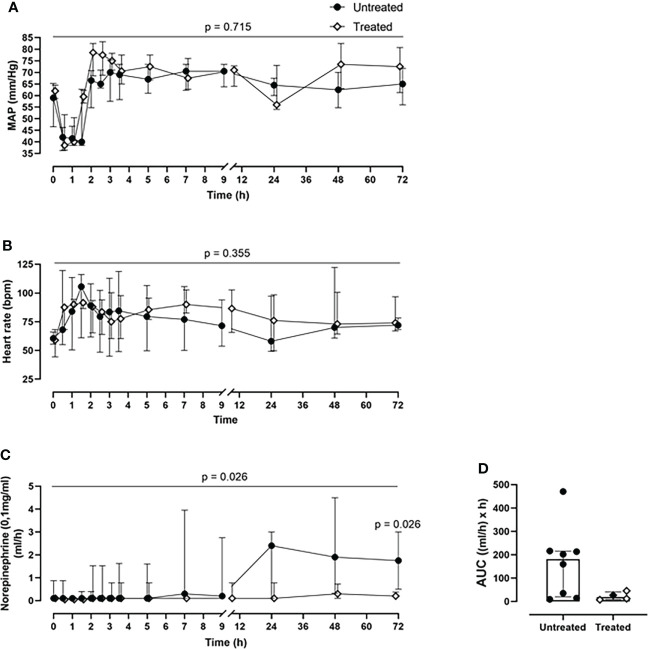
*Cardiovascular monitoring.* **(A)** MAP and **(B)** heart rate monitoring. **(C)** Norepinephrine dosing monitoring and the corresponding **(D)** AUC analysis revealed that the treated group (n=4) required less norepinephrine for maintaining the MAP≥60mmHg, compared to the untreated group (n=8). Overall p-value for group comparison of treated vs. untreated animals over time is displayed above the summary trajectories. In the AUC graphical representation, the first pig, receiving lower inhibitor dosage, is represented as black filled diamond.

The cardiovascular monitoring did not show any significant differences in MAP and heart rate in the pairwise group comparison per time point (**Figures 2A, B**), or in the longitudinal mixed model analysis (p=0.716 for MAP, p=0.355 for heart rate). Nonetheless, during the entire experiment, the treated group responded better to the resuscitation therapy, while requiring less norepinephrine for positive ionotropic support therapy compared with the untreated group (p=0.026, mixed model analysis). Therefore, the norepinephrine requirements to maintain the MAP at ≥60mmHg could be reduced by 88% as deduced from the AUC in the treated group, with a statistically significant reduction of the norepinephrine dosage at 72-h timepoint post-injury (p=0.026) (**Figures 2C, D**).

### Combined C5 and CD14 inhibition therapy mitigated the systemic inflammation

**Figure 3:**
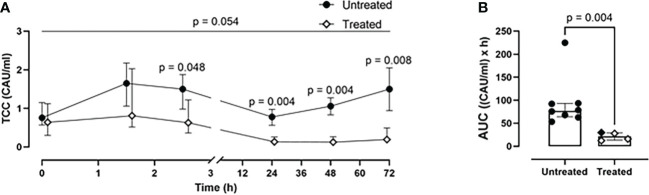
*Blood TCC measurements.* **(A)** TCC formation 2.5-, 24-, 48-, and 72-h after trauma was reduced in the treated group (n=4), compared to the untreated group (n=8). **(B)** Area under the curve analysis showed a statistically significant reduction of TCC in the treated group. Overall p-value for group comparison of treated vs. untreated animals over time is displayed above the summary trajectories. In the AUC graphical representation, the first pig, receiving lower inhibitor dosage, is represented as black filled diamond.

A time-dependent generation of TCC was observed in the untreated group (**Figure 3**). This was significantly reduced in the treated group at 2.5- (p=0.048), 24- (p=0.004), 48- (p=0.004), and 72-h (p=0.008) (**Figure 3A**). Overall, the combined blockade therapy effectively reduced complement activation as indicated by significantly reduced TCC formation by 70% (p= 0.004) as measured by AUC throughout the 72-h of the experiment (**Figure 3B**).

**Figure 4:**
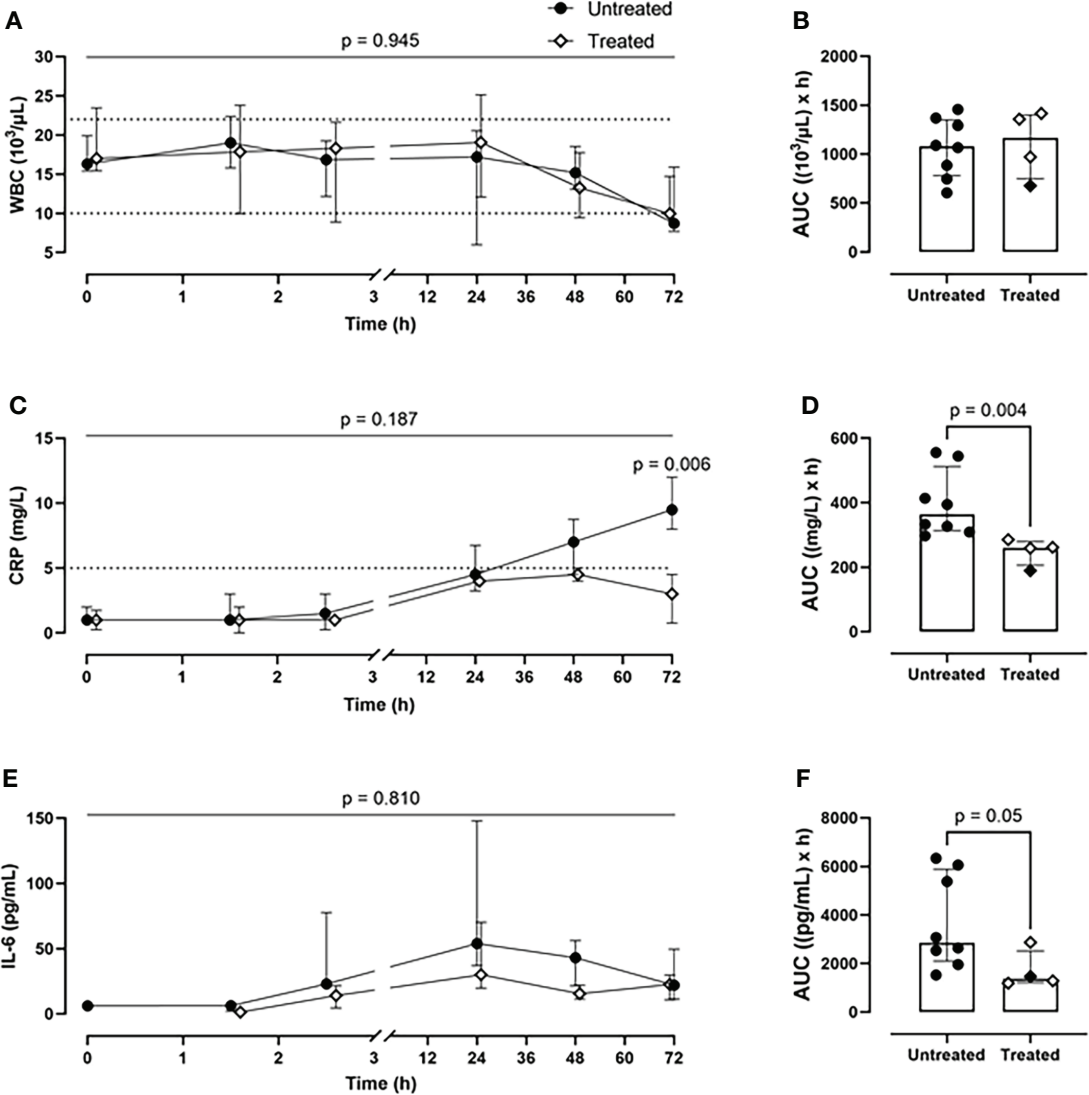
*Systemic inflammatory parameters.* **(A)** WBCs monitoring and **(B)** WBCs AUC analysis showed no difference between the two groups. **(C)** CRP monitoring and **(D)** CRP AUC analysis revealed a significant reduction of CRP values in blood in the treated group (n=4), compared to the untreated group (n=8). **(E)** IL-6 monitoring and **(F)** IL-6 AUC analysis showed a diminished IL-6 expression in the treated group (n=4), compared to the untreated group (n=8). Dotted lines represent the normal reference range. Overall p-value for group comparison of treated vs. untreated animals over time is displayed above the summary trajectories. In the AUC graphical representation, the first pig, receiving lower inhibitor dosage, is represented as black filled diamond.

Although the inhibition therapy did not alter the WBC count (**Figures 4A, B**), it significantly ameliorated the signs of the posttraumatic cytokine storm. The therapy achieved a systemic reduction of the key inflammatory parameters: CRP by 28% (p=0.004) (**Figures 4C, D**) and IL-6 by 52% (p=0.05) (**Figures 4E, F**). The therapy had no effect on blood TNF level (**Supplementary Figures 2A, B**).

### Combined C5 and CD14 inhibition therapy prevented the PLT consumption and TAT generation

**Figure 5:**
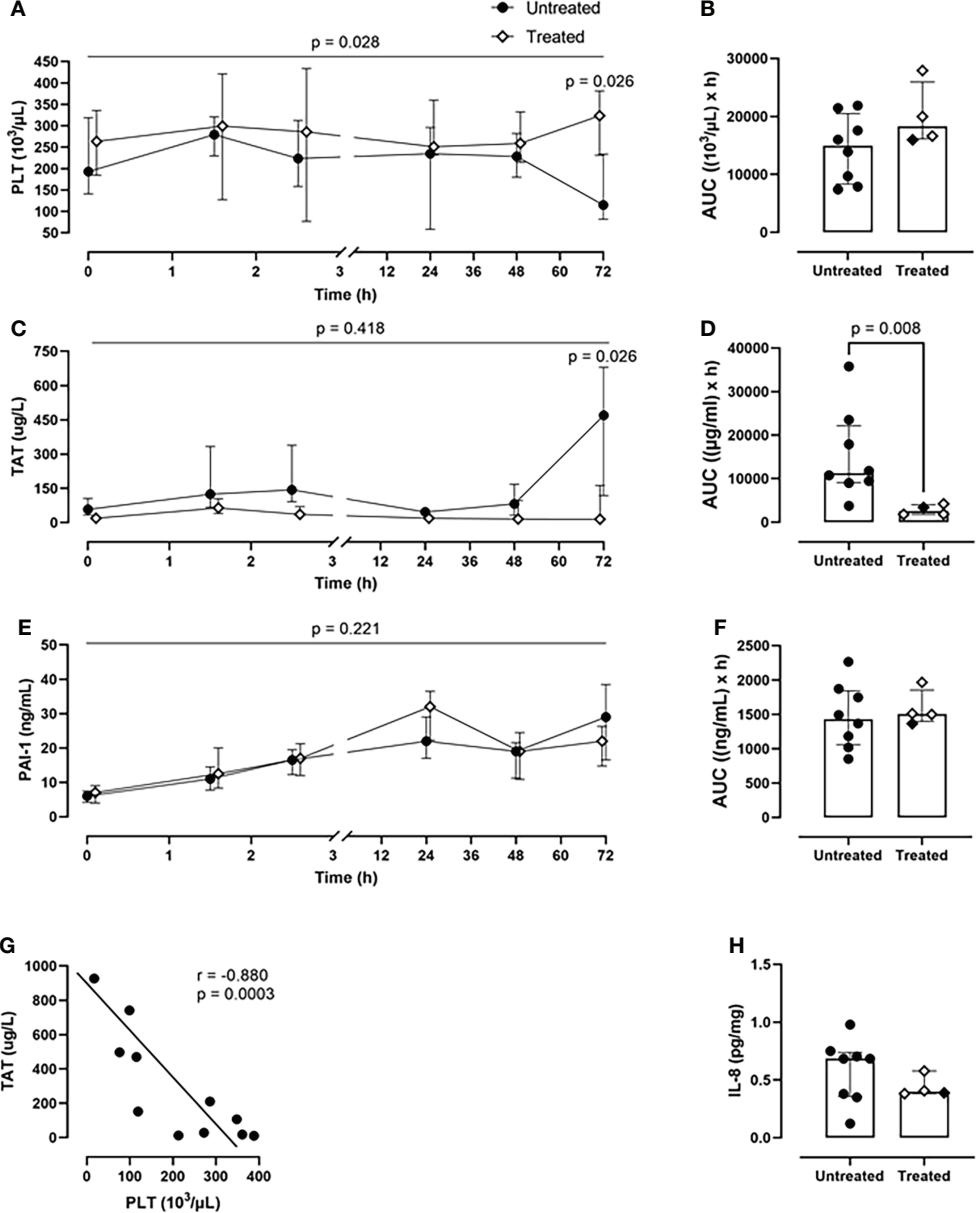
*Coagulation parameters.* **(A)** PLT count monitoring and **(B)** PLT count AUC analysis revealed a reduced PLTs consumption in the treated group (n=4), compared to the untreated group (n=8). **(C)** TAT monitoring and **(D)** TAT AUC analysis showed a significant reduction in TAT complexes formation in the treated group (n=4), but not in the untreated group (n=8). **(E)** PAI-1 monitoring and **(F)** PAI-1 AUC did not differ between the two groups. **(G)** Spearman correlation analysis shows a negative statistically significant correlation between TAT values and PLTs count in blood 72-h post-injury. **(H)** IL-8 measurement in BALF showed a reduction of the IL-8 levels in the treated group (n=4) compared to the untreated group (n=8). Overall p-value for group comparison of treated vs. untreated animals over time is displayed above the summary trajectories. In the AUC graphical representation, the first pig, receiving lower inhibitor dosage, is represented as black filled diamond.

Over the course of 72-h of monitoring, the treated group showed a greater PLT count (p=0.028, mixed model analysis) and a significant reduction of PLT consumption at the 72-h timepoint (p=0.026) in comparison with the untreated group (**Figure 5A**). Overall, the combined inhibitory strategy diminished the systemic PLT consumption by 18% during the experiment (**Figure 5B**). The therapy strongly reduced the TAT formation by 77% (p=0.008) as deduced from AUC (**Figures 5C, D**). The combined therapy did not affect PAI-1 concentrations (**Figures 5E, F**).

A significant negative correlation was detected between PLT consumption and TAT formation (r=−0.880; p=0.0003) in both groups (**Figure 5G**).

Furthermore, the treated group showed lower IL-8 values in BALF compared to the untreated group (**Figure 5H**).

### Combined C5 and CD14 inhibition therapy protected against organ dysfunction

**Figure 6:**
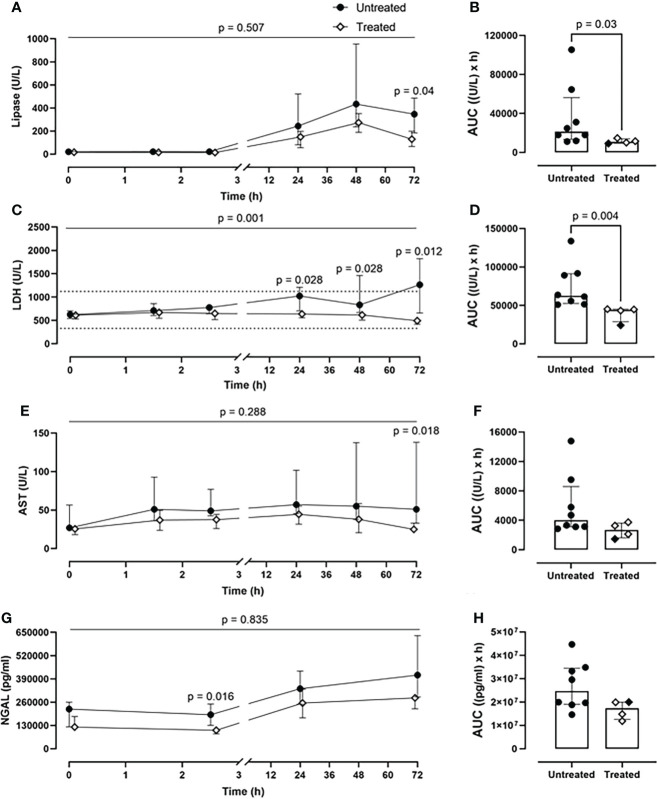
*Organ damage markers.* **(A)** Lipase, **(C)** LDH, **(E)** AST, and **(G)** NGAL monitoring in addition to **(B)** lipase, **(D)** LDH, **(F)** AST, and **(H)** NGAL AUC analysis showed that significantly lower lipase, LHD, AST, and NGAL values were detected in blood in the treated group (n=4), compared to the untreated group (n=8). Dotted lines represent the normal reference range. Overall p-value for group comparison of treated vs. untreated animals over time is displayed above the summary trajectories. In the AUC graphical representation, the first pig, receiving lower inhibitor dosage, is represented as black filled diamond.

The combined inhibitory therapy was associated with a lower amount of plasma markers reflecting organ damage. The unspecific lipase concentrations were significantly reduced in the treated group at 72-h timepoint (p=0.04) (**Figure 6A**). Overall, the lipase values were reduced by 50% in the treated group (p=0.03) as seen in the AUC measurement (**Figure 6B**). Substantially lower LDH blood values were detected in the treated compared to the untreated group over 72-h of experiment (p=0.001, mixed model analysis) and specifically at 24- (p=0.028), 48-(p=0.028), and 72-h timepoint (p=0.012) (**Figure 6C**). Globally, in the treated group, the LDH plasma concentrations were significantly reduced by 30% (p=0.004) (**Figure 6D**). The therapy reduced the AST values by 33%, with a significant reduction at 72-h (p=0.018) (**Figures 6E, F**). The treated group displayed a significant decrease in the kidney damage marker NGAL plasma concentrations of 30%, with a significant reduction at 2.5-h post-injury (p=0.016) (**Figures 6G, H**).

### Combined C5 and CD14 inhibition therapy prevented tissue C5b-9 deposition

**Figure 7:**
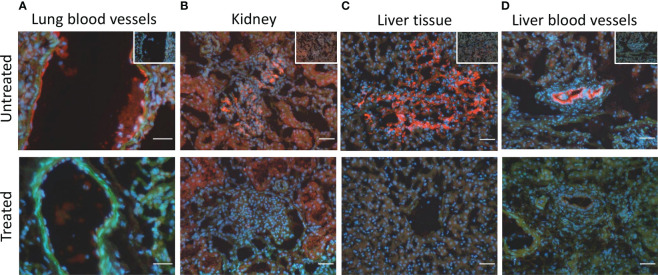
*C5b-9 immunofluorescent staining* showed C5b-9 deposition (red) **(A)** on the blood vessels in lung tissue in 5/7 of the untreated animals and in none of the four treated animals. **(B)** In kidney tissue, C5b-9 glomerular deposition was detected in 5/7 untreated and in 2/4 treated animals. **(C)** In the liver tissue, C5b-9 deposition was detected in 5/7 untreated and in none if the treated animals. **(D)** C5b-9 deposition was also detected in the liver blood vessels in the untreated but not in the treated group. One representative section from each group is shown. To facilitate the recognition of the histological structures the nuclear counterstaining (blue) and F-actin counterstaining (green) have been performed. In the upper panel, in the right upper corner, the respective ‘no primary antibody controls’ are illustrated. Magnification bars (short white lines bottom right in all panels): 20µm (lung) and 50µm (kidney and liver).

The immunofluorescent staining of the lung tissue revealed the deposition of C5b-9 on blood vessels in five of the seven animals in the untreated group that lived for 72-h, but in none of the four animals in the treated group (p=0.06) (**Figure 7A**). In kidney tissue, C5b-9 glomerular deposition was detected in five from seven untreated animals and in two from four untreated animals (**Figure 7B**). In the liver tissue, C5b-9 deposition was detected in five from seven untreated animals and in none of the treated animals (p=0.06) (**Figure 7C**). Moreover, C5b-9 deposition on the liver blood vessels was detected in the untreated compared to the treated animals (**Figure 7D**).

## Discussion

The present study reports the efficacy of a combined C5 and CD14 inhibition therapy in an exploratory study of a long-term large animal polytrauma, with standard surgical and intensive case management. We demonstrated that drug administration given 30 min postinjury strongly attenuated the trauma-induced pro-inflammatory response by diminishing the systemic CRP and IL-6 expression. 30 minutes is within a clinically relevant window since treatment can be given by prehospital personnel if the trauma does not occur close enough to a hospital. Furthermore, the combined therapy successfully prevented classical signs of posttraumatic coagulopathy by reducing the PLT consumption and the formation of TAT complexes. The significant negative correlation between the PLT consumption and TAT formation mirrors the crosstalk among hemostasis, complement and the TLR system, as shown in previous studies that in trauma settings an increased amount of thrombin is generated by PLTs upon TLR2 and TLR4 activation (34, 35).

Importantly, the treatment reduced organ damage and the need for norepinephrine. The analysis of the organ damage markers clearly showed that the inhibition therapy protected the liver, kidneys, and lungs, minimizing the risk for developing MODS. Thus, the treated group displayed lower blood lipase, LDH, and AST values compared to the untreated group. Moreover, double inhibition therapy reduced C5b-9 deposition in the liver tissue and blood vessels. In the treated group, lower NGAL levels were detected. NGAL as an early marker of acute kidney injury (36), displays an upregulated expression upon TLR stimulation (37). Therefore, blocking CD14, required for several TLRs signaling, can inhibit NGAL overexpression and prevent trauma related acute kidney injury (TRAKI) (8). The double inhibition therapy reduced the C5b-9 deposition in kidney glomeruli, thus minimizing the risk of developing TRAKI. Elevated levels of TCC in plasma of trauma patients were shown to be associated with an increased risk of developing acute lung injury (7). By inhibition of the complement system at the C5 level, we obtained a substantial reduction of TCC in plasma, and a complete abolishment of C5b-9 deposition in the lung blood vessels of the treated animal group. Previously, the polytrauma pig model used in our study was proven to be reliable and clinically relevant for investigating long-term pulmonary consequences of polytrauma (26). In the study conducted by Horst *et al.*, IL-8 measurements in BALF revealed a significant increase of IL-8 at 72-h in the polytrauma group compared to the sham group (26). In the present study, we showed that the combined inhibition therapy strongly reduces the IL-8 values in the BALF in the polytrauma treated compared to the polytrauma untreated group, thus limiting the local pulmonary inflammation.

Substances targeting the complement system or TLR signaling pathways have long been considered potential therapies for various diseases (28, 38). The effectiveness of the complement inhibition therapies at the C3 level (compstatin) and C5 level (RA101295), along with CD14 inhibition monotherapy were already proven to be beneficial in porcine and baboon sepsis models (39–43). In these studies, the complement or CD14 inhibition attenuated the sepsis-induced inflammation and coagulopathy (42, 43), displayed protective effects on the endothelium, and was associated with improved organ performance (39, 40). While the complement or CD14 inhibition monotherapy proved to be effective, the combined inhibition therapy displayed clear enhanced effects. In a polymicrobial sepsis mouse model, the C5 or CD14 inhibition monotherapy strongly reduced the overall inflammation but failed to increase the survival rates, whereas the combined inhibition therapy significantly reduced the inflammation and increased the survival (22), hence proving its therapeutic advantage. A study of experimental porcine sepsis reported an enhanced efficacy of the combined C5 and CD14 inhibition in limiting the thrombo-inflammation and hemodynamic instability over single monotherapies (21). These results were validated in another study of porcine polymicrobial sepsis, where the combined inhibition also significantly improved the survival rates (25). Moreover, the therapeutic efficacy of the C5 and/or CD14 therapy was assessed in a well-established human *ex vivo* whole blood model of inflammation, where it was shown that the combined inhibition therapy exceeded the effects of monotherapies in limiting the inflammation (23, 24). Considering the strong evidence of combined inhibition superiority over the monotherapies, in this study we addressed only the combined inhibition therapy.

Additionally, clinical trials were conducted for efficacy analysis of anti-C5a monoclonal antibody (CaCP29, InflaRx GmbH) (NCT02246595) and of an MD2-TLR4 antagonist (Eritoran, Eisai) in severe sepsis (NCT00334828), but without any clinical effect. We have shown in an *in vitro* human whole blood model that CD14 was substantially more effective in attenuating *Escherichia coli*- and *Staphylococcus aureus*-induced inflammatory reactions than the specific TLR4 inhibitor Eritoran, supporting a broader effect of inhibition of the co-receptor CD14 than that of Eritoran, consistent with CD14 being a co-receptor for several TLRs, including TLR4 and TLR2 (23).

Numerous studies have reported long-term immune dysfunction after sepsis, surgical events, as well as after Covid-19 infection (44, 45), with monocyte exhaustion syndrome being one of the underlying causes (46, 47). Exhausted monocytes are shown to be skewed into the classical monocytes’ population (murine CD11b^+^CD115^+^Ly6C^high^, human CD14^+^CD16^-^HLA-DR^mid^) (18, 47), and are characterized by cellular NAD^+^ depletion, elevated ROS production, and impaired response to secondary stimuli (47, 48). Mechanistically, exhausted monocytes display an exacerbated activation of signal transducer and activator of transcription 1 (STAT1) and Kruppel-like factor 4 (KLF4). STAT1 involvement in monocytes polarization towards classical and intermediate pro-inflammatory subsets was also confirmed using machine learning methods (16). STAT1 as well as KLF4 can be triggered by interferon-γ (IFN-γ), which will induce a pro-inflammatory cellular response (49, 50). Complement and/or CD14 inhibition successfully reduced the IFN-γ production in mice and baboons’ sepsis studies (22, 43), thus preventing STAT1 and KLF4 overactivation. Additionally, interferon gamma-induced protein 10 (IP-10), which expression is dependent on STAT1 signaling (51), was significantly reduced in heart, liver, spleen, and kidney by the combined inhibition therapy in porcine *Escherichia coli* sepsis model (30). Using single-cell RNA-sequencing technique it was showed that persistent low dosed of LPS could induce the generation of two pro-inflammatory subset of monocytes: one subset with a high expression of C5aR1 and interferon activated genes, resembling the human intermediate monocyte subset, and the second subset with high CCR2 expression, resembling the human classical monocyte subset (16). Interestingly, in another study LPS-exhausted monocytes were reposted to increase the expression programmed death-ligand 1 (PD-L1) exhaustion marker, which could be prevented by C5aR1 inhibition (15). TLR4 as well as TLR2 (52) adaptor TRAM was also shown to play a role in monocyte exhaustion generation (47, 53). As CD14 is a co-receptor of TLR2 and TLR4 (9), its inhibition will prevent the overactivation of TRAM to a greater extent than single TLR inhibition. Indeed, in human whole blood model, *Escherichia coli*-induced upregulation of CD11b on monocytes was stronger reduced by CD14 inhibitor alone or in combination with complement inhibition than by a specific TLR4 inhibitor (23). Thus, directly though inhibition of CD14, or indirectly through reduction of cytokine production and release, CD14 inhibition together with complement inhibition can prevent the monocyte exhaustion syndrome and its long-term immune consequences.

Beyond sepsis research, complement antagonists are considered potential therapeutic strategies in severe cases of Covid-19 infections (54, 55). Therefore, ongoing clinical trials are investigating the effectiveness of a C3 inhibitor (AMY-101, Amyndas Pharmaceuticals S.A., PA) in Covid-19-induced acute respiratory distress syndrome (NCT04395456), and of a C5 inhibitor (Zilucoplan, UCB Pharma, Brussels, Belgium) in Covid-19-induced respiratory failure (NCT04382755). Furthermore, several clinical trials are being conducted to assess anti-CD14 monoclonal antibody (IC14, Implicit Bioscience, WA, USA) in patients with Covid-19 infection (NCT04346277; NCT04391309).

The timing rationale of the combined C5 and CD14 inhibition therapy application was based on the data from previous studies (56–58). The complement and coagulation system is activated within minutes after trauma in polytraumatized patients, with the first signs of trauma-induced coagulopathy and complement consumption developing within the first 74 min post-injury (56, 57). The estimated onset of MODS immunopathology was reported to be in the first hours after a traumatic insult (58). The dysregulated immune response post-injury is aggravated by the early total care approach, for example intramedullary nailing surgical strategy (2), as performed in our study. Consequently, it is vital to initiate the immunomodulatory therapy in the first “golden” hour after trauma. Previous studies using the combined C5 and CD14 therapy in pigs undergoing sepsis have been based on the “proof of concept” principle where the drug was given before bacteria administration (20). An *in vitro* study using whole human blood incubated with bacteria and inhibition of complement and CD14 showed that post-challenge inhibition as compared to pre-challenge also attenuated the inflammatory response reasonably well during the first period of time, but the effect was gradually lost (24). In the present study, we took advantage of the clinically relevant therapeutic strategy of post-challenge blocking the immune response, but still before reaching the “point of no return”. This timing principle has been previously proposed as an “upstream approach” (59).

In the present study, the low n-size supports the 3Rs’ principles (replacement, reduction, refinement), but could be accompanied with a potential risk for statistical errors. Nevertheless, this did not weaken the robustness of the data, as the difference between the groups was substantial. The unequal sample sizes, due to drug supply shortages, are a limitation of the study. Nevertheless, it was carefully approached in the statistical analysis of the data. Because of the real-time pharmacodynamic analysis, the investigators could not be blinded, and due to drug availability issues, the animals could not be randomized. The performed meticulous monitoring of the pharmacodynamics of C5 and CD14 inhibitors allowed a prompt adjustment of the dosages for accomplishing the study goals. Although the first treatment animal received a lower dosage of the C5 and CD14 inhibitors, its pharmacological efficacy was comparable with the adjusted therapy regimen during the experiments, which included higher dosages of inhibitors and these four animals could therefore be combined as the treatment group. These findings justify the patient-tailored therapeutic approaches. Because the immune response of a multiple trauma patient is unpredictable, it requires close continuous monitoring, with a consequent adjustment of the therapy. The fact that most of the readouts were statistically significant with only four pigs in the treatment group and eight in the control group, underscores the substantial significance between the groups and that type I errors are highly unlikely. However, we cannot exclude that there were some type II errors.

In summary, we demonstrated that the early administration of the combined blockade of C5 and CD14 post-injury, adjusted in response to subsequent immune monitoring, significantly reduced the catecholamine requirements, thrombo-inflammatory response, and organ damage in a 72-h porcine polytrauma model. These data have important implications for future therapeutic strategies in trauma.

## Data availability statement

The raw data supporting the conclusions of this article will be made available by the authors, without undue reservation.

## Ethics statement

The animal study was reviewed and approved by Ministry for Nature, Environment and Consumer Protection in North Rhine-Westphalia, Recklinghausen, Germany; AZ 81-02.04. 2020.A215.

## Author contributions

MH-L, TM, FH, JG, MG, and KH designed the animal study. JG, ÜM, and LL performed the animal study. JG, ÜM, LL, KQ, XZ, AP, and YL collected the data. LL, JL, KP, and CL performed the experiments. BM, TM, and LL analyzed and interpreted the data. LL drafted the manuscript. All authors critically revised the manuscript and approved the final version.

## Funding

This research was funded by the Deutsche Forschungsgemeinschaft (DFG, German Research Foundation) – 429837092. RA101295 was provided by UCB Pharma (Brussels, Belgium).

## Acknowledgments

The authors would like to thank the Flow Cytometry core facility of University Clinic Aachen, especially Nathalie Steinke and Silke Vaßen for valuable support.

## Conflict of interest

The authors declare that the research was conducted in the absence of any commercial or financial relationships that could be construed as a potential conflict of interest.

## Publisher’s note

All claims expressed in this article are solely those of the authors and do not necessarily represent those of their affiliated organizations, or those of the publisher, the editors and the reviewers. Any product that may be evaluated in this article, or claim that may be made by its manufacturer, is not guaranteed or endorsed by the publisher.
