# Supplementary material for: Simultaneous C5 and CD14 inhibition limits inflammation and organ dysfunction in pig polytrauma
Source: Front Immunol. 2022 Aug 18;13:952267. doi: 10.3389/fimmu.2022.952267 (PMC9433645; doi:10.3389/fimmu.2022.952267)
Supplement: Supplementary file 1 [file DataSheet_1.pdf]

Suppl. Fig. 1

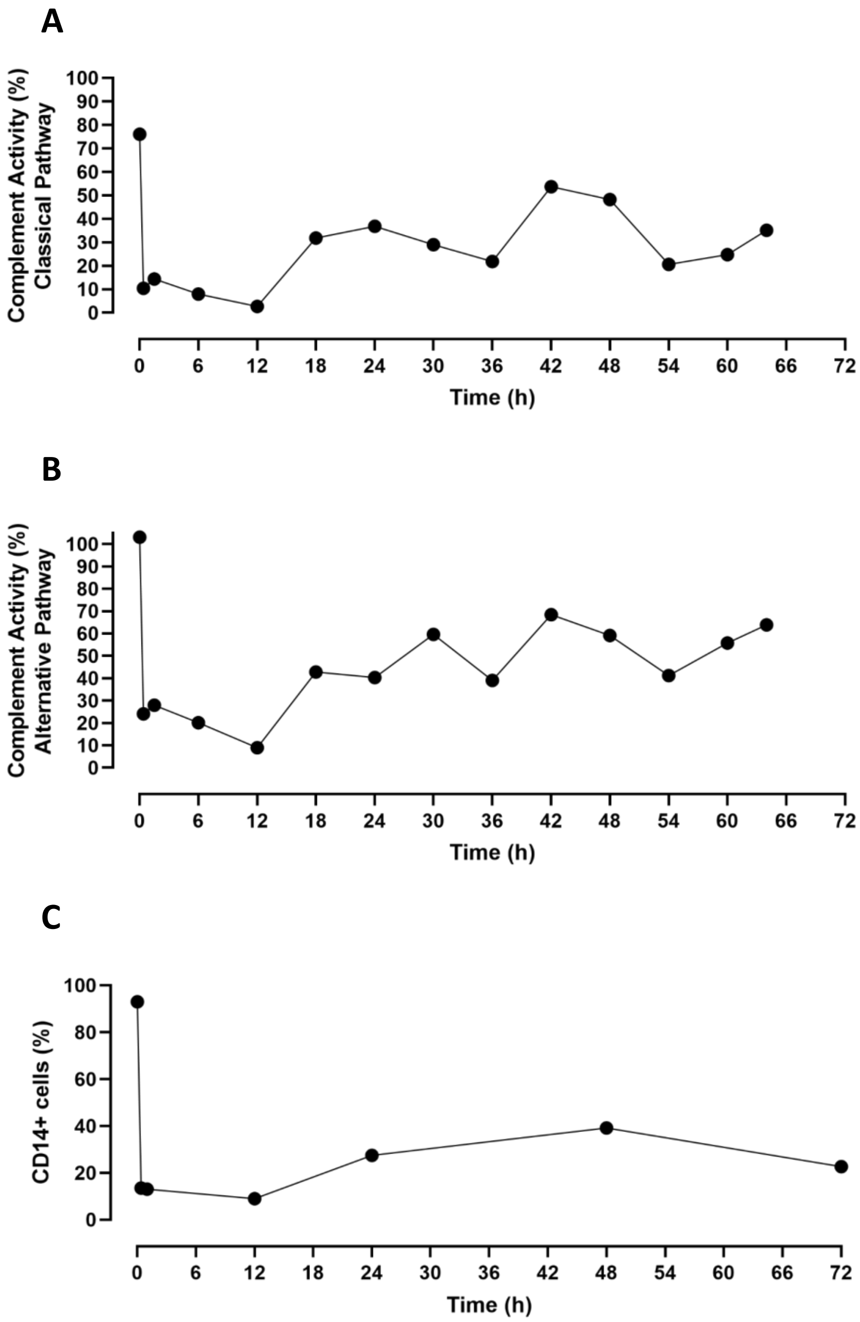

Suppl. Fig. 1. Pharmacodynamic analysis of the preliminary regimen of C5 and CD14 inhibitory therapy. The C5 inhibitor RA101295 (3mg/kg bolus and 0.55mg/kg/h continuous infusion) blocked the classical pathway of the complement system by 78% (A), and the alternative pathway by 61% (B). rMil2 (one bolus of 5mg/kg) ensured the CD14 antigen saturation of 82% (C); n=1.

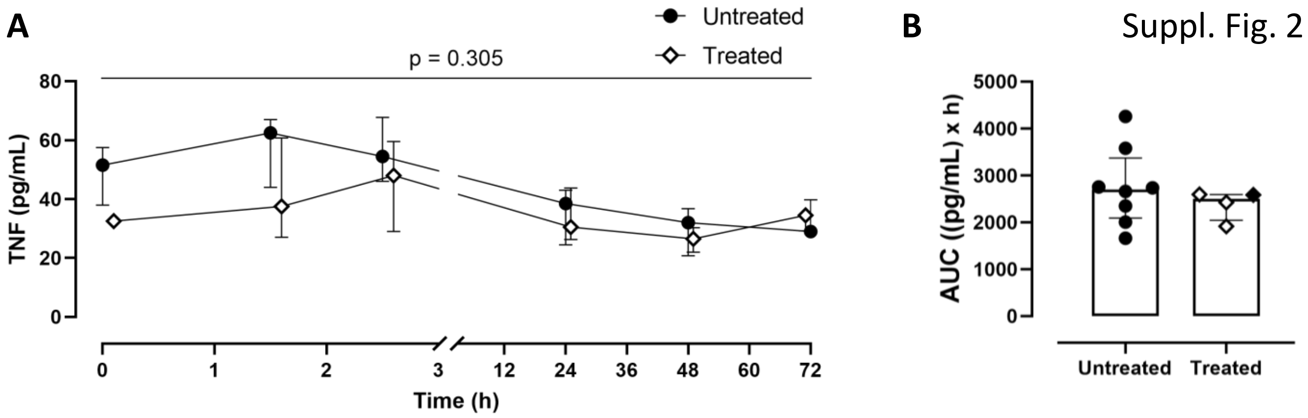

Supp. Fig 2. The TNF and ALT measurements in plasma. (A) The TNF values were slightly increased in the untreated group (n=8) compared to the double blockade treated group (n=4); (B) the TNF AUC analysis showed no difference between the groups. Data is shown as median and interquartile range.
